# Stathmin Regulates Hypoxia-Inducible Factor-1**α** Expression through the Mammalian Target of Rapamycin Pathway in Ovarian Clear Cell Adenocarcinoma

**DOI:** 10.1155/2013/279593

**Published:** 2013-05-30

**Authors:** Kazuhiro Tamura, Mikihiro Yoshie, Eri Miyajima, Mika Kano, Eiichi Tachikawa

**Affiliations:** Department of Endocrine and Neural Pharmacology, Tokyo University of Pharmacy & Life Sciences, Horinouchi 1432-1, Hachioji, Tokyo 192-0392, Japan

## Abstract

Stathmin, a microtubule-destabilizing phosphoprotein, is highly expressed in ovarian cancer, but the pathophysiological significance of this protein in ovarian carcinoma cells remains poorly understood. This study reports the involvement of stathmin in the mTOR/HIF-1**α**/VEGF pathway in ovarian clear cell adenocarcinoma (CCA) during hypoxia. HIF-1**α** protein and VEGF mRNA levels were markedly elevated in RMG-1 cells, a CCA cell line, cultured under hypoxic conditions. Rapamycin, an inhibitor of mTOR complex 1, reduced the level of HIF-1**α** and blocked phosphorylation of ribosomal protein S6 kinase 1 (S6K), a transcriptional regulator of mTOR, demonstrating that hypoxia activates mTOR/S6K/HIF-1**α** signaling in CCA. Furthermore, stathmin knockdown inhibited hypoxia-induced HIF-1**α** and VEGF expression and S6K phosphorylation. The silencing of stathmin expression also reduced Akt phosphorylation, a critical event in the mTOR/HIF-1**α**/VEGF signaling pathway. By contrast, stathmin overexpression upregulated hypoxia-induced HIF-1**α** and VEGF expression in OVCAR-3 cells, another CCA cell line. In addition, suppression of Akt activation by wortmannin, a phosphoinositide 3-kinase (PI3K) inhibitor, decreased HIF-1**α** and VEGF expression. These results illustrate that regulation of HIF-1**α** through the PI3K/Akt/mTOR pathway is controlled by stathmin in CCA. Our findings point to a new mechanism of stathmin regulation during ovarian cancer.

## 1. Introduction 

Most ovarian cancers are believed to arise from epithelial cells residing on the outer surface of the ovary. Histologically, human ovarian cancers are classified as serous cyst, clear cell (CCA), and endometrioid adenocarcinomas [1–3]. CCA accounts for 20% of ovarian cancers and 25% of all surface epithelial tumors. Because no symptoms are present during early stages of ovarian cancer, its diagnosis is usually delayed. This has contributed to an increase in the number of individuals with CCA in Japan. CCA is also resistant to chemotherapy; thus, it associates with a poor prognosis. 

Tumors often express vascular endothelial growth factor (VEGF) in response to local hypoxia [3, 4]. VEGF expression is an indicator of angiogenesis, and cancer cell proliferation and invasion [3–7]. Increased VEGF expression can also stimulate neovascularization and contribute to tumor growth. VEGF expression in different tissues is regulated by hypoxia inducible factor (HIF)-1*α* [8]. Hypoxia inhibits the hydroxylation of HIF-1*α* and its subsequent proteasomal degradation, resulting in the translocation of HIF-1*α* into the nucleus and in the transcription of numerous genes, including VEGF [9–11]. The phosphatidylinositol 3-kinase (PI3K) signaling pathway modulates HIF-1*α* protein levels. PI3K activates many downstream molecules via Akt, and PI3K signaling is involved in several aspects of tumorigenesis [1, 12]. For example, Akt phosphorylates numerous substrates, including the mammalian target of rapamycin (mTOR; it is a component of two complexes, mTORC1 and mTORC2), a master regulator of protein translation. mTORC1 controls translation via two major substrates, ribosomal protein S6K (S6K) and 4E-BP1 [13]. Recent studies have implicated mTOR in several human diseases, including ovarian cancer [10, 14, 15]. Other studies have reported that the mTOR pathway is activated in ovarian cancer cells [16, 17]. Furthermore, treatment with everolimus, an analogue of rapamycin, lowered the levels of phosphorylated mTOR (p-mTOR), HIF-1*α*, and VEGF [18].

Stathmin, a cytosolic phosphoprotein, plays an important role in regulating the dynamics of microtubules (MTs), cytoskeletal components involved in cell shape, motility, and division [19]. Stathmin depolymerizes MTs by either sequestering free tubulin dimers or directly inducing MT-catastrophe, and it is involved in cell differentiation [20, 21] and migration [22]. Stathmin may also be essential for cancer cell survival [23]. More recently, stathmin has been shown to associate with PI3K activity in ovarian cancer [24], supporting the hypothesis that stathmin may be linked to the progression of ovarian cancer. In a previous study, we reported stathmin knockdown to inhibit the activation of Akt and HIF-1*α* in human endometrial and endothelial cells [25]; however, there is no study on the involvement of the PI3K/Akt/mTOR pathway and stathmin in HIF expression during hypoxia in cultured CCA cells. In this study, we investigated the role of stathmin in the mTOR/HIF-1*α*/VEGF pathway when CCA cells were cultured under hypoxic conditions. 

## 2. Materials and Methods

### 2.1. Reagents

The mTOR inhibitor, rapamycin, was purchased from Sigma-Aldrich (St. Louis, MO, USA). The PI3K inhibitor, wortmannin, was obtained from AppliChem (Darmstadt, Germany). These reagents were dissolved in culture medium containing 0.05% (v/v) ethanol. Recombinant EGF was obtained from R&D Systems, Inc. (Minneapolis, MN, USA). A monoclonal HIF-1*α* antibody was purchased from BD Biosciences (Oxford, UK). Polyclonal phospho-S6K, S6K, phospho-Akt (ser-473, p-Akt), and total Akt antibodies were from Cell Signaling Technology (Beverly, MA, USA). A monoclonal *β*-actin antibody was purchased from Sigma-Aldrich. A polyclonal stathmin antibody was kindly donated by Dr. Andre Sobel (Institut du Fer a Moulin, Paris, France). Horseradish peroxidase-labeled goat anti-mouse and anti-rabbit IgG antibodies (secondary antibodies) were from Vector Laboratories (Burlingame, CA, USA). 

### 2.2. Ovarian Carcinoma Cell Culture and Hypoxic Condition

Human CCA cell lines, RMG-1 and OVCAR-3, were cultured in Dulbecco's modified Eagle and RPMI media (Wako Pure Chemical Industries Ltd., Osaka, Japan) supplemented with 10% (v/v) charcoal-stripped fetal bovine serum (FBS; HyClone, South Logan, UT, USA), 50 *μ*g/mL penicillin, 50 *μ*g/mL streptomycin, 100 *μ*g/mL neomycin, and 0.5 *μ*g/mL amphotericin B (Life Technologies, Carlsbad, CA, USA). Hypoxic conditions were generated with AnaeroPack, a chemical agent that absorbs oxygen and generates carbon dioxide (Mitsubishi Gas Chemical Co., Tokyo, Japan) in a square chamber, according to the manufacturer's instructions. This apparatus maintained hypoxic conditions of 0.1–1% (v/v) O_2_ and 5% (v/v) CO_2_, as previously reported [26]. 

### 2.3. Immunoblotting

Cells were lysed with RIPA buffer (Cell Signaling Technology) according to the manufacturer's instructions. Equal amounts (10 *μ*g) of protein were separated by SDS-PAGE and electrophoretically transferred to polyvinylidene difluoride membranes (Millipore, Billerica, MA, USA). Membranes were blocked with 5% (w/v) nonfat milk and incubated with different antibodies overnight at 4°C. Immunoreactive bands were detected by enhanced chemiluminescence (PerkinElmer Life Science, Inc., Boston, MA, USA) after incubation with an appropriate secondary antibody (0.5 *μ*g/mL). Membranes were treated with stripping solution (25 mM glycine-HCl (pH 2.0) containing 1% (w/v) SDS) and reprobed with another antibody. 

### 2.4. RNA Extraction and Analysis by Real-Time and Semi-Quantitative RT-PCR

Total RNA was isolated with Isogen (Nippon Gene, Tokyo, Japan) according to the manufacturer's instructions. RNA (100 ng) was amplified by real-time RT-PCR using the iScript One-Step RT-PCR Kit with SYBR Green (Bio-Rad Laboratories, Hercules, CA, USA). Reactions were carried out on an iQ5 Real-Time PCR Detection System (Bio-Rad). Sense (S) and antisense (AS) primers were 5′-GCTACTGCCATGACC-3′ (S) and 5′-ATGGACTCGCACATC-3′ (AS) for VEGF (GenBank Accession No. AB021221) and 5′-AGCCACATCGCTCAGACA-3′ (S) and 5′-GCCCAATACGACCAAATCC-3′ (AS) for glyceraldehyde-3-phosphate dehydrogenase (GAPDH) (GenBank Accession No. AF261085). Fold change in the expression of each gene was calculated using the ΔΔCt method with GAPDH as an internal control [26]. 

### 2.5. Knockdown of Stathmin with Small Interfering RNA (siRNA)

RMG-1 or OVCAR-3 cells in 24-well culture plates at approximately 60% confluency were transfected with either nontargeting control (50 nM, AllStar Negative control; QIAGEN, Mississauga, ON, Canada) or stathmin (50 nM, sc-36127; Santa Cruz Biotechnology, Santa Cruz, CA, USA) siRNAs using Lipofectamine RNAiMAX transfection reagent (Invitrogen) according to the manufacturer's instructions. Stathmin siRNA targeted three different regions of the 3′-untranslated mRNA sequence. After transfection for 24 h, the medium was removed, and cells were cultured with fresh medium for an additional 24 h. Cells were then cultured for 5 h under normoxic or hypoxic conditions. The concentration of siRNA to be used in knockdown studies was carefully titrated in pilot experiments.

### 2.6. Preparation of the Stathmin Expression Vector and Transfection

The pTriEx-3 expression system (Novagen, Palo Alto, CA, USA) was used to examine the effect of induced stathmin expression in OVCAR-3 cells. In this experiment, we used OVCAR-3 cells because stathmin could not be overexpressed in RMG-1 cells with this system. Briefly, the open-reading frame of the human stathmin gene (GenBank Accession no. J049911.1) was amplified by PCR and subcloned into the pTriEx-3 vector. Subconfluent cells in 12-well dishes were transfected with the vector (125 ng per well) using GeneJuice transfection reagent (Novagen). OVCAR-3 cells transfected with empty pTriEx-3 vector served as the control. The pTriEx-3-stathmin-transfected cells were cultured under normoxic or hypoxic conditions.

### 2.7. Statistical Analysis

Each experiment was repeated three times, and results were reproducible. Results from mRNA expression experiments are presented as means ± SEM. Significance was assessed by the Tukey-Kramer multiple comparisons test. A *P*  value <0.05 was considered statistically significant.

## 3. Results

### 3.1. Hypoxia-Induced mTOR/HIF-1*α*/VEGF Signaling and the Effect of Stathmin Knockdown on the Activation of the mTOR Signaling Pathway in CCA Cell Lines

**Figure 1:**
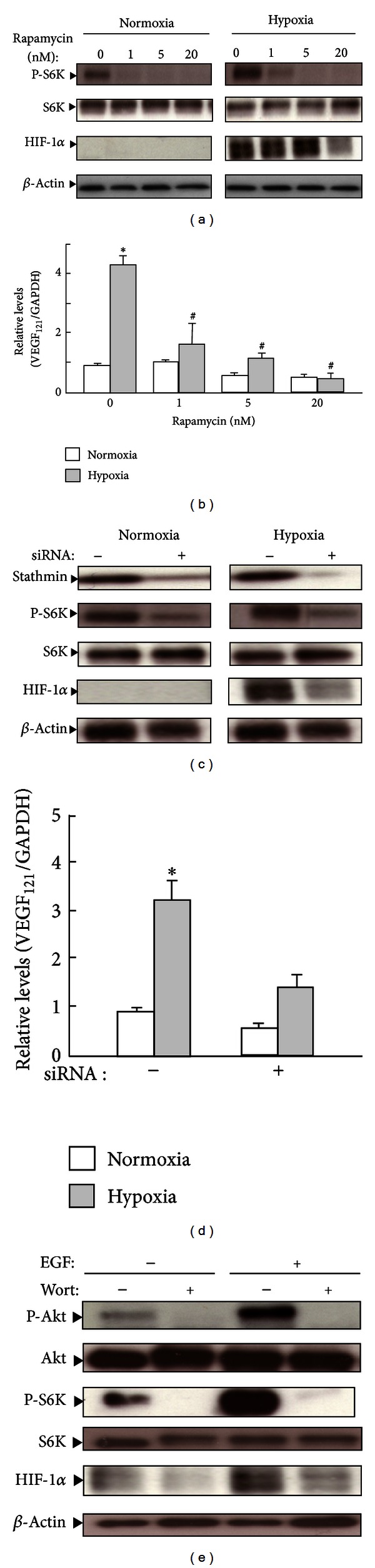
Effects of rapamycin (a) and stathmin knockdown (c) on the phosphorylation of S6K and the expression of HIF-1*α* and VEGF in RMG1 cells cultured under hypoxic conditions. ((a) and (b)) Cells were treated with 1–20 nM rapamycin and then cultured under normoxic or hypoxic conditions for 5 h. ((c) and (d)) Cells were pretreated for 24 h with control (–) or stathmin (+) siRNAs and then cultured under normoxic and hypoxic conditions for 5 h. (e) Cells were pretreated with or without a PI3K inhibitor, wortmannin (100 nM) for 30 min, and then cultured under hypoxic conditions for 5 h in the presence or absence of EGF. (a, c, and e) Cell lysates were subjected to immunoblot analysis for phosphorylated S6 K (p-S6K), S6K, HIF-1*α*, phosphorylated Akt (p-Akt), total Akt (Akt), or *β*-actin. The same blot in each panel was probed, stripped, and then reprobed with each antibody. The levels of *β*-actin, S6K, and Akt were used as loading controls. Representative data from three independent experiments are shown. ((b) and (d)) Total RNA was extracted and subjected to real-time quantitative RT-PCR to analyze VEGF_121_ mRNA expression. Data from three independent experiments are shown as ratios of the control level during normoxia, and results are shown as mean ± SEM. *P < 0.05  versus normoxia. ^#^ *P* < 0.05  versus no treatment.

To determine whether mTOR/HIF-1*α* signaling participates in hypoxia, RMG-1 cells were treated with an mTOR inhibitor, rapamycin, under normoxic and hypoxic conditions. The levels of phosphorylated S6K (p-S6K) (Figure 1(a)) and HIF-1*α* increased under hypoxic conditions compared to normoxic conditions (Figure 1(a)). Rapamycin treatment also reduced the level of phosphorylated S6K at the doses between 1 and 20 nM and markedly decreased the hypoxia-induced HIF-1*α* protein level at 20 nM. Real-time RT-PCR analysis showed a 4.4-fold increase in VEGF_121_ expression during hypoxia but not during normoxia, and rapamycin at 1, 5, and 20 nM reduced VEGF_121_ levels dose-dependently (Figure 1(b)). Furthermore, exposure of cells to increasing concentrations of rapamycin (1–50 nM) for 24 h did not affect cell viability (data not shown). Based on these results, we concluded the mTOR signaling pathway to regulate hypoxia-induced HIF-1*α*/VEGF expression in RMG-1 cells; thus, the effects of stathmin knockdown on S6K phosphorylation and HIF-1*α* and VEGF expression were examined (Figures 1(c) and 1(d)). Stathmin knockdown clearly decreased phosphorylated S6K in both normoxic and hypoxic groups, while the level of total S6K was unchanged. Furthermore, stathmin siRNA reduced hypoxia-induced expression of HIF-1*α*(Figure 1(c)) and VEGF (Figure 1(d)). In results shown in Figures 1(a) and 1(c), S6K phosphorylation was observed in cells cultured under normoxic conditions, and marked increases in hypoxia-induced S6K phosphorylation was not seen. Following the addition of recombinant EGF, which activated the PI3K/Akt signaling cascade through the EGF receptor, the levels of p-Akt, p-S6K, and HIF-1*α* increased under hypoxic conditions in the absence of wortmannin (Figure 1(e)). By contrast, the expression of these genes was inhibited by wortmannin. These data indicate that EGF stimulates HIF-1*α* protein expression in part through the activation of Akt and S6K.

### 3.2. Effect of Stathmin Overexpression and Knockdown on Hypoxia-Induced HIF-1*α* and VEGF Expression in OVCAR-3 Cells

**Figure 2:**
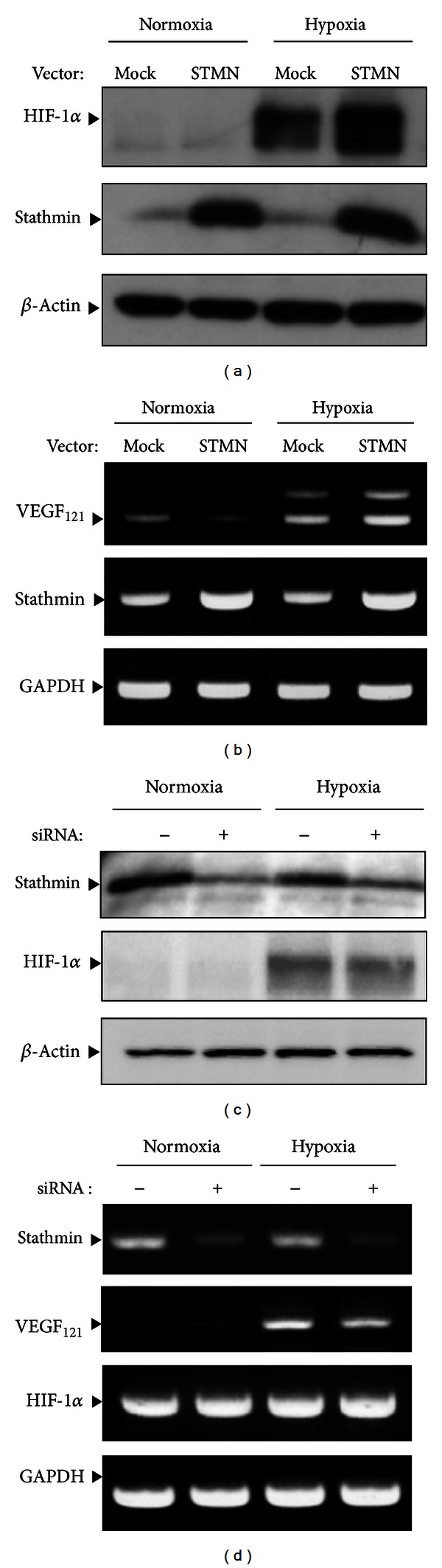
Effects of stathmin overexpression ((a) and (b)) and knockdown ((c) and (d)) on hypoxia-induced HIF-1*α* and VEGF expression in OVCAR-3 cells.(a) Cells were transfected with an empty (Mock) or stathmin expression (STMN) vector and then cultured under hypoxic conditions for 5 h. (b) Cells were treated with control (–) or stathmin (+) siRNA and then cultured under normoxic or hypoxic conditions for 5 h. ((a) and (c)) Cell lysates were subjected to immunoblot analysis for HIF-1*α* and stathmin. The same blot was probed, stripped, and then reprobed with each antibody. The level of *β*-actin was used as a loading control. ((b) and (d)) Total RNA was extracted and subjected to semiquantitative RT-PCR to analyze stathmin, VEGF, and HIF-1*α* mRNA expression. Representative data from three independent experiments are shown.

To examine the involvement of stathmin in the regulation of HIF-1*α* expression in CCA, another ovarian CCA cell line, OVCAR-3, was transfected with pTriEx-stathmin vector (STMN) or its empty vector (Mock) (Figure 2(a)). Stathmin overexpression was observed in STMN-transfected cells. The hypoxia-induced HIF-1*α* level in STMN-treated cells was compared with those in mock-treated cells (Figure 2(a)). An increase in HIF-1*α* protein was observed in stathmin overexpressed cells under hypoxic conditions. As shown in Figure 2(b), stathmin overexpression upregulated VEGF expression. By contrast, the levels of HIF-1*α* (Figure 2(c)) and VEGF (Figure 2(d)) decreased after stathmin siRNA. There was no change in HIF-1*α* expression in stathmin siRNA-transfected cells compared with the control. 

### 3.3. Effects of Stathmin Knockdown on Hypoxia-Induced Akt Phosphorylation and HIF-1*α* Expression and Effects of a PI3K Inhibitor on HIF-1*α* and VEGF in CCA

**Figure 3:**
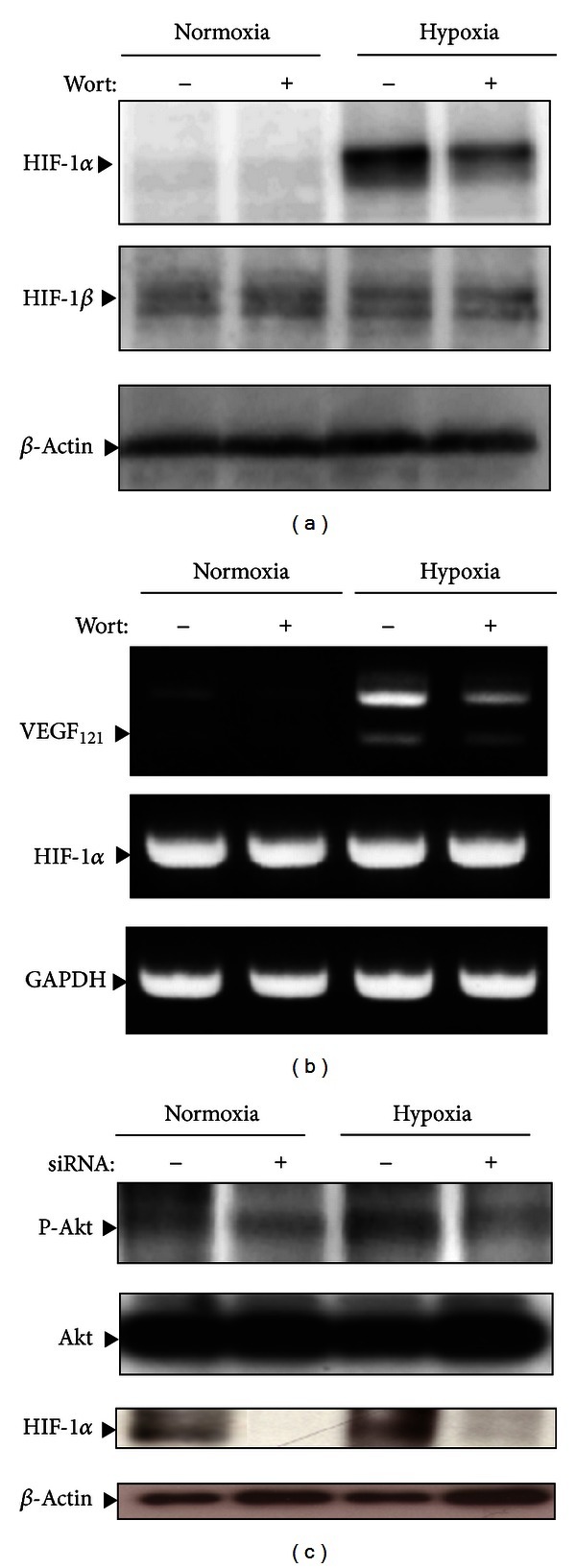
Effects of PI3K inhibitor ((a) and (b)) and stathmin knockdown (c) on hypoxia-induced HIF-1*α* and VEGF expression in OVCAR-3 cells. ((a) and (b)) Cells were pretreated without (–) or with (+) a PI3K inhibitor, wortmannin (100 nM), for 30 min and then cultured under hypoxic conditions for 5 h. Cell lysates and total RNAs were subjected to immunoblot analysis (a, HIF-1*α*, HIF-1*β*, and *β*-actin) and semiquantitative RT-PCR analysis (b, VEGF and HIF-1*α*), respectively. Representative data from three independent experiments are shown. (c) Cells were treated with control (–), or stathmin (+) RNAi and then cultured under normoxic or hypoxic conditions for 5 h. Cell lysates were subjected to immunoblot analysis for phosphorylated p-Akt (p-Akt), total Akt (Akt), or HIF-1*α*. Representative data from three independent experiments are shown.

To further determine whether hypoxia-induced HIF-1*α* expression is mediated through the Akt pathway in OVCAR-3 cells and further whether stathmin expression alters the PI3K/Akt/HIF-1*α* signaling, the levels of HIF-1*α* were investigated after pretreatment with wortmannin under normoxic and hypoxic conditions (Figure 3). Wortmannin decreased HIF-1*α* during hypoxia (Figure 3(a)). Furthermore, wortmannin suppressed hypoxia-induced VEGF mRNA expression without changing the HIF-1*α* mRNA level (Figure 3(b)). Based on these results, which indicated a link between PI3K/Akt signaling and HIF-1*α*, we investigated the effects of stathmin knockdown on Akt activation. Stathmin knockdown decreased Akt phosphorylation during both normoxia and hypoxia, but the level of total Akt was unchanged (Figure 3(c)). Thus, stathmin regulates HIF-1*α* via the PI3K/Akt signaling pathway in CCA.

## 4. Discussion 

The microenvironment of a tumor is closely linked to cell proliferation, invasion, metastasis, and chemotherapeutic resistance [2, 27]. The inner environment of solid cancerous masses is often characterized by low oxygen and pH and insufficient nutrients because of inadequate circulation. Tumor angiogenesis and lymphangiogenesis are important features of tumor progression and metastasis [26]. Misregulated angiogenesis due to rapid proliferation of tumorigenic cells may result in acute and chronic ischemia, especially in the center of solid ovarian tumors. Low oxygen can upregulate different genes, and this can affect malignancy and chemotherapeutic resistance. HIF-1*α*, an important transcriptional factor, promotes tumor angiogenesis by upregulating different angiogenic genes [8–11]. HIF-1*α* is also highly expressed in female-specific cancers such as ovarian cancer [5, 28]. A previous study reported higher VEGF expression in ovarian cancer than in normal ovarian tissue. VEGF is an HIF-1*α*-driven angiogenic factor involved in cancer cell proliferation and invasion [28]. Bevacizumab, a humanized antibody against human VEGF, has been used in the treatment of ovarian cancer [2, 29]. Elevated HIF-1*α* and VEGF levels positively correlated with poor prognosis in ovarian cancer patients [29]. To the best of our knowledge, the present work provides the first evidence that hypoxia upregulates HIF-1*α* and VEGF expression in CCA partially through activation of the PI3 K/Akt/mTOR signaling pathway. Our data illustrate that changes in the HIF-1*α* protein level were not due to transcriptional regulation because there was no significant change in HIF-1*α* mRNA expression (data not shown). Furthermore, we observed that hypoxia-induced VEGF mRNA levels were reduced by rapamycin, an inhibitor of mTORC1. 

In addition, higher expression of the EGF receptor or its activated mutant has been detected in ovarian cancer patients [30]. Activated PI3K and Akt can induce cell transformation through S6K phosphorylation [31]. S6K activation also correlates with ovarian cancer [32]. Treatment with a PI3K inhibitor suppressed the phosphorylation of Akt and S6K, an mTORC1 effector, and HIF-1*α* expression. Exogenous EGF increased the levels of Akt, S6K, and HIF-1*α* during hypoxia. Moreover, we observed effective suppression of EGF-stimulated Akt/S6K activity and VEGF expression by KU0063794, an inhibitor of both mTORC1 and mTORC2 (data not shown). mTORC2 is presumed to phosphorylate Akt [33]. Taken together, these results show that Akt is an upstream regulator of the mTOR/S6K/HIF-1*α* signaling pathway and that this pathway is triggered by both hypoxia and EGF. Thus, stimulation of the EGF receptor in CCA, which may be directly associated with the progression of ovarian cancer, can potentiate the PI3K/Akt/mTOR pathway.

Stathmin is expressed abundantly in a variety of human cancers, and stathmin overexpression is an indicator of poor prognosis in ovarian cancer [34]. The expression of p27kip, a cell-cycle regulatory protein that interacts with cyclin-CDK2 and CDK4, inhibits cell-cycle progression at G1 and is inversely related to stathmin expression [35]. Stathmin knockdown decreased S6K phosphorylation as well as HIF-1*α* and VEGF expression in CCA, suggesting that stathmin regulates hypoxia-induced HIF-1*α* and VEGF expression through the mTOR/S6K pathway. We previously demonstrated that stathmin knockdown decreased MT depolymerization and the HIF-1*α* protein level in endothelial and endometrial cells [26]. Consistent with the above report, the present results suggest that altered MT dynamics may modulate HIF-1*α* and VEGF expression in cancer cells. Because 2-methoxy-estradiol destabilizes MT at doses that are effective in inhibiting tumor growth and angiogenesis* in vivo* [36], there might be a mechanistic link between disruption of the MT cytoskeleton and inhibition of angiogenesis [37]. Furthermore, eliminating resistance to existing anticancer drugs is critical for effective chemotherapy, and stathmin has been found to profoundly influence drug sensitivity [38–42]. For example, stathmin silencing reduced resistance of cancer cells to anticancer drugs that perturbed MT dynamics. In another gene profiling study involving cisplatin-resistant and -sensitive ovarian tissues, stathmin was described as a cisplatin resistance-related protein [38]. Elevated stathmin expression has also been observed in paclitaxel-resistant ovarian cancer cell lines where it enhanced resistance by preventing tubulin polymerization and promoting MT destabilization and disassembly [39]. Others have speculated that stathmin may decrease the binding affinity of MT inhibitors [42]. While stathmin silencing reduced the proliferation of gastric carcinoma cells [43], targeted therapies that disrupt stathmin function are still under investigation. At this point, additional studies are needed to clarify the involvement of stathmin in MT dynamics and angiogenesis, and analysis of patient samples might provide important insights. In this study, we found mTOR inhibition and stathmin knockdown to inhibit angiogenic responses during hypoxia, which may be potentiated by local growth factors. Further studies are warranted to understand better the mTOR/P70S6K pathway in the context of MT dynamics and ovarian cancer. 

## Abbreviations

HIF-1: Hypoxia inducible factor-1
mTOR: Mammalian target of rapamycin
S6K: Ribosomal protein S6 kinase 1
(VEGF): Vascular endothelial growth factor
(GAPDH): Glyceraldehyde-3-phosphate dehydrogenase.

## References

[1] Romero I, Bast RC (2012). Minireview: human ovarian cancer: biology, current management, and paths to personalizing therapy. *Endocrinology*.

[2] Campos SM, Ghosh S (2010). A current review of targeted therapeutics for ovarian cancer. *Journal of Oncology*.

[3] Sudo T (2012). Molecular-targeted therapies for ovarian cancer: prospects for the future. *International Journal of Clinical Oncology*.

[4] Folkman J (1971). Tumor angiogenesis: therapeutic implications. *New England Journal of Medicine*.

[5] Fujimoto J, Sakaguchi H, Hirose R, Ichigo S, Tamaya T (1998). Biological implication of the expression of vascular endothelial growth factor subtypes in ovarian carcinoma. *Cancer*.

[6] Bandiera E, Franceschini R, Specchia C (2012). Prognostic significance of vascular endothelial growth factor serum determination in women with ovarian cancer. *ISRN Obstetrics and Gynecology*.

[7] Cheng D, Liang B, Li Y (2013). Serum vascular endothelial growth factor (VEGF-C) as a diagnostic and prognostic marker in patients with ovarian cancer. *PLoS ONE*.

[8] Ahluwalia A, Tarnawski AS (2012). Critical role of hypoxia sensor-HIF-1*α* in VEGF gene activation. Implications for angiogenesis and tissue injury healing. *Current Medicinal Chemistry*.

[9] Ivan M, Kondo K, Yang H (2001). HIF*α* targeted for VHL-mediated destruction by proline hydroxylation: implications for O_2_ sensing. *Science*.

[10] Harris AL (2002). Hypoxia—a key regulatory factor in tumour growth. *Nature Reviews Cancer*.

[11] Fokas E, McKenna WG, Muschel RJ (2012). The impact of tumor microenvironment on cancer treatment and its modulation by direct and indirect antivascular strategies. *Cancer and Metastasis Reviews*.

[12] Cheung M, Testa JR (2013). Diverse mechanisms of AKT pathway activation in human malignancy. *Current Cancer Drug Targets*.

[13] Labplante M, Sabatini DM (2012). mTOR signaling in growth control and disease. *Cell*.

[14] Sengupta S, Peterson TR, Sabatini DM (2010). Regulation of the mTOR Complex 1 Pathway by Nutrients, Growth Factors, and Stress. *Molecular Cell*.

[15] Banerjee S, Kaye SB (2013). New strategies in the treatment of ovarian cancer: current clinical perspectives and future potential. *Clinical Cancer Research*.

[16] Miyazawa M, Yasuda M, Fujita M (2009). Therapeutic strategy targeting the mTOR-HIF-1*α*-VEGF pathway in ovarian clear cell adenocarcinoma. *Pathology International*.

[17] Miyazawa M, Yasuda M, Fujita M (2010). Granulosa cell tumor with activated mTOR-HIF-1*α*-VEGF pathway. *Journal of Obstetrics and Gynaecology Research*.

[18] Trinh XB, Tjalma WAA, Vermeulen PB (2009). The VEGF pathway and the AKT/mTOR/p70S6K1 signalling pathway in human epithelial ovarian cancer. *British Journal of Cancer*.

[19] Curmi PA, Gavet O, Charbaut E (1999). Stathmin and its phosphoprotein family: general properties, biochemical and functional interaction with tubulin. *Cell Structure and Function*.

[20] Iancu-Rubin C, Gajzer D, Tripodi J (2011). Down-regulation of stathmin expression is required for megakaryocyte maturation and platelet production. *Blood*.

[21] Yoshie M, Kashima H, Bessho T, Takeichi M, Isaka K, Tamura K (2008). Expression of stathmin, a microtubule regulatory protein, is associated with the migration and differentiation of cultured early trophoblasts. *Human Reproduction*.

[22] Liu F, Sun YL, Xu Y, Liu F, Wang LS, Zhao XH (2013). Expression and phosphorylation of stathmin correlate with cell migration in esophageal squamous cell carcinoma. *Oncology Reports*.

[23] Belletti B, Baldassarre G (2011). Stathmin: a protein with many tasks. New biomarker and potential target in cancer. *Expert Opinion on Therapeutic Targets*.

[24] Karst AM, Levanon K, Duraisamy S (2011). Stathmin 1, a marker of PI3K pathway activation and regulator of microtubule dynamics, is expressed in early pelvic serous carcinomas. *Gynecologic Oncology*.

[25] Yoshie M, Miyajima E, Kyo S, Tamura K (2009). Stathmin, a microtubule regulatory protein, is associated with hypoxia-inducible facto Ma levels in human endometrial and endothelial cells. *Endocrinology*.

[26] Gomes FG, Nedel F, Alves AM, Nör JE, Tarquinio SB (2013). Tumor angiogenesis and lymphangiogenesis: tumor/endothelial crosstalk and cellular/microenvironmental signaling mechanisms. *Life Sciences*.

[27] Hida K, Akiyama K, Ohga N, Maishi N, Hida Y (2013). Tumour endothelial cells acquire drug resistance in a tumour microenvironment. *The Journal of Biochemistry*.

[28] Chen Y, Zhang L, Pan Y, Ren X, Hao Q (2012). Over-expression of semaphorin4D, hypoxia-inducible factor-1*α* and vascular endothelial growth factor is related to poor prognosis in ovarian epithelial cancer. *International Journal of Molecular Sciences*.

[29] Carpini JD, Karam AK, Montgomery L (2010). Vascular endothelial growth factor and its relationship to the prognosis and treatment of breast, ovarian, and cervical cancer. *Angiogenesis*.

[30] Cheng JC, Klausen C, Leung PC (2013). Hypoxia-inducible factor 1 alpha mediates epidermal growth factor-induced down-regulation of E-cadherin expression and cell invasion in human ovarian cancer cells. *Cancer Letters*.

[31] Aoki M, Blazek E, Vogt PK (2001). A role of the kinase mTOR in cellular transformation induced by the oncoproteins P3k and Akt. *Proceedings of the National Academy of Sciences of the United States of America*.

[32] Ip CK, Wong AS (2012). Exploiting p70 S6 kinase as a target for ovarian cancer. *Expert Opinion on Therapeutic Targets*.

[33] Rodrik-Outmezguine VS, Chandarlapaty S, Pagano NC (2011). mTOR kinase inhibition causes feedback-dependent biphasic regulation of AKT signaling. *Cancer Discovery*.

[34] Nemunaitis J (2012). Stathmin 1: a protein with many tasks, new biomarker and potential target in cancer. *Expert Opinion on Therapeutic Targets*.

[35] Iancu-Rubin C, Atweh GF (2005). p27Kip1 and stathmin share the stage for the first time. *Trends in Cell Biology*.

[36] Mabjeesh NJ, Escuin D, LaVallee TM (2003). 2ME2 inhibits tumor growth and angiogenesis by disrupting microtubules and dysregulating HIF. *Cancer Cell*.

[37] Escuin D, Kline ER, Giannakakou P (2005). Both microtubule-stabilizing and microtubule-destabilizing drugs inhibit hypoxia-inducible factor-1*α* accumulation and activity by disrupting microtubule function. *Cancer Research*.

[38] Gong F, Peng X, Zeng Z, Yu M, Zhao Y, Tong A (2011). Proteomic analysis of cisplatin resistance in human ovarian cancer using 2-DE method. *Molecular and Cellular Biochemistry*.

[39] Balachandran R, Welsh MJ, Day BW (2003). Altered levels and regulation of stathmin in paclitaxel-resistant ovarian cancer cells. *Oncogene*.

[40] Tan HT, Wu W, Ng YZ (2012). Proteomic analysis of colorectal cancer metastasis: stathmin-1 revealed as a player in cancer cell migration and prognostic marker. *Journal of Proteome Research*.

[41] Martello LA, Verdier-Pinard P, Shen HJ (2003). Elevated levels of microtubule destabilizing factors in a Taxol-resistant/dependent A549 cell line with an *α*-tubulin mutation. *Cancer Research*.

[42] Su D, Smith SM, Preti M (2009). Stathmin and tubulin expression and survival of ovarian cancer patients receiving platinum treatment with and without paclitaxel. *Cancer*.

[43] Kang W, Tong JH, Chan AW (2012). Stathmin1 plays oncogenic role and is a target of microRNA-223 in gastric cancer. *PLoS ONE*.

